# Dynamic causal modeling reveals increased cerebellar- periaqueductal gray communication during fear extinction

**DOI:** 10.3389/fnsys.2023.1148604

**Published:** 2023-05-17

**Authors:** Elena Paci, Bridget M. Lumb, Richard Apps, Charlotte L. Lawrenson, Rosalyn J. Moran

**Affiliations:** ^1^School of Physiology, Pharmacology and Neuroscience, University of Bristol, Bristol, United Kingdom; ^2^Department of Neuroimaging, King’s College London, London, United Kingdom

**Keywords:** cerebellum, periaqueductal gray, fear, dynamic causal modeling (DCM), ERPs, extinction

## Abstract

**Introduction:**

The extinction of fear memories is an important component in regulating defensive behaviors, contributing toward adaptive processes essential for survival. The cerebellar medial nucleus (MCN) has bidirectional connections with the ventrolateral periaqueductal gray (vlPAG) and is implicated in the regulation of multiple aspects of fear, such as conditioned fear learning and the expression of defensive motor outputs. However, it is unclear how communication between the MCN and vlPAG changes during conditioned fear extinction.

**Methods:**

We use dynamic causal models (DCMs) to infer effective connectivity between the MCN and vlPAG during auditory cue-conditioned fear retrieval and extinction in the rat. DCMs determine causal relationships between neuronal sources by using neurobiologically motivated models to reproduce the dynamics of post-synaptic potentials generated by synaptic connections within and between brain regions. Auditory event related potentials (ERPs) during the conditioned tone offset were recorded simultaneously from MCN and vlPAG and then modeled to identify changes in the strength of the synaptic inputs between these brain areas and the relationship to freezing behavior across extinction trials. The DCMs were structured to model evoked responses to best represent conditioned tone offset ERPs and were adapted to represent PAG and cerebellar circuitry.

**Results:**

With the use of Parametric Empirical Bayesian (PEB) analysis we found that the strength of the information flow, mediated through enhanced synaptic efficacy from MCN to vlPAG was inversely related to freezing during extinction, i.e., communication from MCN to vlPAG increased with extinction.

**Discussion:**

The results are consistent with the cerebellum contributing to predictive processes that underpin fear extinction.

## Introduction

The cerebellum is associated with emotional control (Snider et al., 1976; Supple et al., 1987; Supple and Leaton, 1990; Ploghaus et al., 2000; Stoodley and Schmahmann, 2010, 2018), and is connected with other brain regions to form networks coordinating fear learning and behavior. There is an array of anatomical and electrophysiological evidence showing that the cerebellum (and in particular the medial cerebellar nuclei, MCN) has reciprocal connections with numerous brain regions associated with emotional processing (Apps and Strata, 2015), including the periaqueductal gray (PAG, Teune et al., 2000; Frontera et al., 2020; Vaaga et al., 2020; Lawrenson et al., 2022), amygdala (Farley et al., 2016; Jung et al., 2022), and prefrontal cortex (PFC, Watson et al., 2016). Although the exact role of the cerebellum in fear processing is still uncertain, evidence suggests that it is involved in the acquisition, expression, consolidation and extinction of a fear memory (Sacchetti et al., 2002, 2005; Koutsikou et al., 2014; Utz et al., 2015; Frontera et al., 2020). A key feature of the cerebellum is that it forms and updates internal models to minimize errors in behavioral performance: if there is a mismatch between predicted and actual experience, a prediction error occurs that is used to update our internal model thus contributing to learning through error driven adaptive processes (Itō, 1984; Wolpert et al., 1998; Ito, 2006). For example, in fear conditioning, the Rescorla-Wagner learning model proposes that prediction error signals from an aversive stimulus is the main driver of learning during the acquisition of associatively conditioned fear memory. In this context the cerebellum is therefore ideally placed to contribute to fear prediction processes (Ernst et al., 2019).

The periaqueductal gray (PAG) is a key area for the regulation of fear behaviors, as well as other survival strategies, and can be divided into functional columns that regulate distinct aspects of fear responses. The ventrolateral region of the PAG (vlPAG) is well known, in rats and mice, for its role in the regulation of fear-related freezing (Vianna et al., 2001; Walker and Carrive, 2003; Tovote et al., 2016; Watson et al., 2016). Moreover, the vlPAG encodes teaching signals and prediction error-like activity, that via feedback circuits with the amygdala, are thought to regulate fear behavior (McNally and Frederick Westbrook, 2006; Walker et al., 2019).

The cerebellum and the vlPAG have reciprocal connections, which in rodents are predominantly contralateral (Teune et al., 2000; Vaaga et al., 2020), while no lateralization of activity has been found in human studies (Cacciola et al., 2019). Our group and others (Frontera et al., 2020; Lawrenson et al., 2022) have demonstrated that the MCN to vlPAG pathway regulates the acquisition and extinction of fear memory as measured by freezing behavior and defensive ultrasonic vocalizations (22 kHz). Activation of the pathway during both fear acquisition or extinction decreases the strength of the fear memory response (Frontera et al., 2020) while inhibition increases fear-related behavioral activity (Frontera et al., 2020; Lawrenson et al., 2022). In addition, neurons within the vlPAG increase their firing frequency in a temporally precise manner in response to the onset and offset of a conditioned tone during fear recall (Lawrenson et al., 2022). Conditioned tone offset related activity in the vlPAG decreases in parallel with fear extinction, and manipulation of cerebellar output during fear consolidation causes a loss in its temporal precision as well as changes in fear behavior (Lawrenson et al., 2022). While the functional significance of such activity remains to be determined, we hypothesize that this is a neural correlate of prediction error, necessary for fear extinction mechanisms, whereby cerebellar projections contribute toward encoding a predictive signal in the PAG.

Previous studies have focused on single unit activity, but event related potentials (ERPs) have also been recorded in response to the tone onset and offset in fear conditioned paradigms (Watson et al., 2016; Lawrenson et al., 2022). Event related potentials (ERPs) are an electrophysiological feature that can be recorded in brain structures in response to events or stimuli (Blackwood and Muir, 1990) and are thought to reveal trial-related predictions errors (Moran R. J. et al., 2013). They represent the summed extracellular activity reflecting the activation of a population of neurons. Moreover, they have been found to be a useful marker in the study of anxiety disorders as differences in the characteristics of the ERPs (such as variability and timing) were identified [e.g., post-traumatic stress disorder (Metzger et al., 1997; Neylan et al., 1999; Skinner et al., 1999)].

In the vlPAG (Lawrenson et al., 2022) the peak amplitude of ERPs has been shown to decrease between early and late extinction trials, while cerebellar ERPs did not exhibit significant extinction-related changes (Lawrenson et al., 2022). Using the same dataset, here we explicitly assess the dynamic interaction between these two structures, and ask whether the ERPs reflect coordinated activity between the MCN and vlPAG during extinction, when the offset responses have appeared.

For this study we have employed a relatively new method to analyze ERPs – known as Dynamic causal modeling (DCM). Initially established in the fMRI field (Friston et al., 2003), this method has been extended to applications on local field potential (LFP) and electroencephalography (EEG) data (Moran et al., 2007; Kiebel et al., 2008). DCM is used to infer effective connectivity, i.e., to understand the effect that a neuronal ensemble in one brain area exerts on another by using Bayesian model inversion. DCMs can be used to determine causal relationships between neuronal sources that rely on neurobiologically motivated models to reproduce the dynamics generated within brain regions (Moran R. et al., 2013). Distinct DCMs have been developed to model different types of generated data, such as evoked, induced and steady-state responses. For this study, DCMs for evoked responses were implemented, to model the tone offset ERPs.

By applying DCMs to the ERPs recorded at the offset of a conditioned tone in the cerebellum and vlPAG we found that there is a predominant influence of the cerebellar to vlPAG pathway in the generation of the ERP responses, and in particular as the strength of the connection increases there is a decrease in freezing behavior. These results indicate that the pathway, via these offset signals, participates in fear extinction.

## Materials and methods

### Animals

All animal procedures were performed in accordance with the UK Animals (Scientific Procedures) Act of 1986 and were approved by the University of Bristol Animal Welfare and Ethical Review Body. Data obtained from a total of six adult male Sprague Dawley rats (280–400 g; Harlan Laboratories) that were used in a previous study (Lawrenson et al., 2022) were analyzed in the present work. The animals were housed under normal environmental conditions in a normal 12 h dark/light cycle and provided with food and water *ad libitum*. Animals were single housed after surgery to prevent damage to implants.

### Surgical procedures for electrophysiological chronic implants

Rats were anaesthetized and mounted in a stereotaxic frame with atraumatic ear bars. Surgery was performed under aseptic conditions, during which craniotomies were performed to gain access to the cerebellum and/or the PAG as required in each line of experiment. Dual microdrives (small drives containing movable tetrodes that can be implanted in animals) were implanted in the medial cerebellar nuclei (MCN, 11.4 mm caudal from bregma, 1 mm lateral from midline, depth of 4 mm) and contralateral ventrolateral periaqueductal gray (vlPAG, 7.5 mm caudal from bregma, 1 mm lateral from midline, depth 4.8 mm). For further details on the surgical procedure see Lawrenson et al. (2022).

### Auditory cued fear conditioning

**FIGURE 1:**
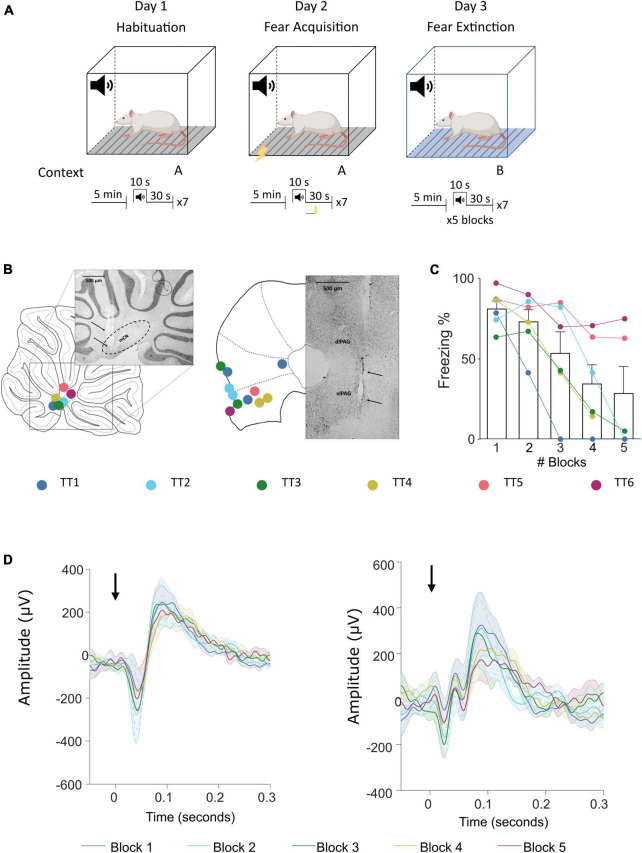
Fear conditioning protocol and ERP recording sites in the cerebellum and vlPAG. **(A)** Diagram of the auditory fear conditioning paradigm composed of habituation, acquisition, and extinction (see Section “Materials and methods” for details). **(B)** Left, histological placement of recording electrodes in the MCN and; right, similar but for the vlPAG. Key, each color represents a separate animal TT1-6. **(C)** Freezing behavior, expressed as percentage, across blocks of extinction; shown as average ± SEM. **(D)** Left, MCN average ± SEM ERP responses recorded during each block (1–5) (*n* = 7, black arrow indicates offset of tone); right, similar but for the vlPAG.

Animals underwent a 3-day auditory cued fear conditioning protocol (Figure 1A; see also Lawrenson et al., 2022). On day 0 animals were habituated to the fear conditioning chamber. On day 1 animals underwent acquisition training and on day 2 retrieval/extinction testing. For all 3 days the tone was delivered at 1 KHz for 10 s. For habituation the animal had a 5 min baseline and then received seven tones with a 30 s intertone interval. For acquisition electrical foot shocks (0.5 s, 0.75 mA) were paired to each of the seven tones and delivered at offset. For extinction 5 × 7 blocks of tones were delivered. For all sessions, videos were recorded with OptiTrack Blackrock software and behavior was manually scored using Solomon Coder software (© 2019 by András Péter). An epoch of at least 2 s where the animal did not move was scored as freezing behavior. At the end of the experiment animals were deeply anaesthetized and terminated by transcardial perfusion (4% paraformaldehyde in 0.1 m phosphate buffer) and the brains extracted to perform histological verification of recording electrode location (Figures 1B, C).

### Neural data analysis

Local field potential (LFP) data were acquired using a Blackrock Microsystems (Utah, USA) data capture system synchronized with OptiTrack software. Neural data were recorded at a sample rate of 30 kHz and post-processed offline. Data was down sampled to 1 kHz and band pass filtered at 1–32 Hz (this was found to be the most appropriate filter to isolate the waveform of the ERPs from other activity/noise) to extract LFPs using MATLAB. Auditory event related potentials (ERPs) were extracted by averaging LFP activity time locked to tone offset using MATLAB (mean based on *n* = 7 trials per animal). In each animal the tetrode recording sites in the MCN and vlPAG yielding, in each case, the largest mean amplitude peak to trough ERP were identified and used to calculate group average data of peak amplitude for each brain site.

### Dynamic causal modeling (connectivity analysis)

Dynamic causal modeling (DCM) was used to infer effective connectivity between the MCN and vlPAG. Here we will first describe the model underlying DCMs for ERPs and then describe some of the key practical information on how to use it.

**FIGURE 2:**
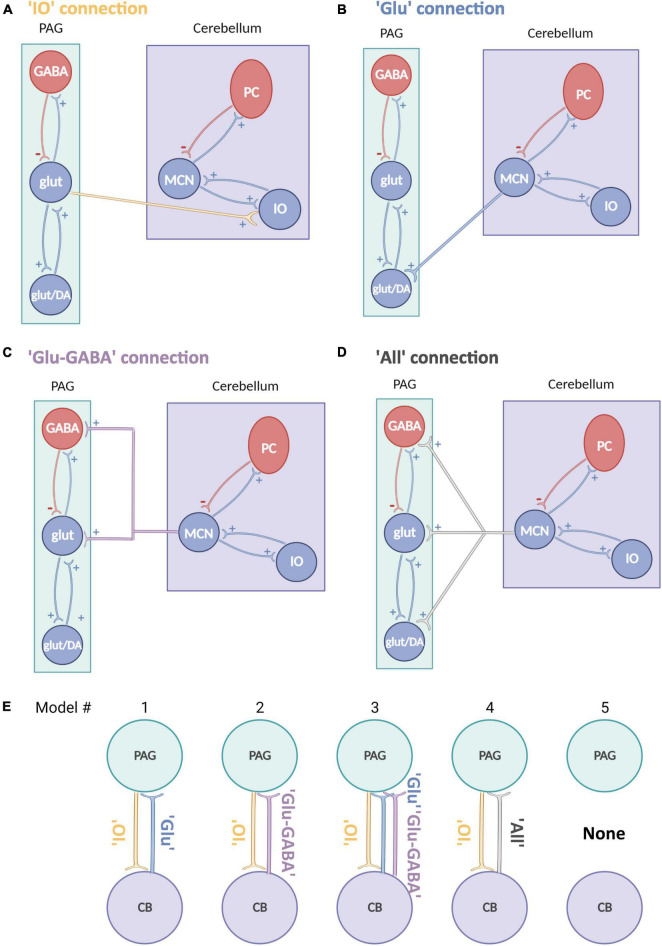
Schematic representation of neural mass models for the cerebellum and PAG. **(A–D)** Schematic neural mass model of the cerebellum (violet box) and vlPAG (green box). Each circle represents a population of neurons. Within the boxes, intrinsic connections are represented in red (inhibitory) and in blue (excitatory). Each panel shows a plausible type of extrinsic connection between the two areas, these are represented between the boxes and synapse on different populations: **(A)** A connection from PAG to cerebellum reaching only glutamatergic neurons (yellow). **(B)** A connection from cerebellum to PAG reaching only an excitatory glutamatergic population (blue). **(C)** A connection from cerebellum to PAG reaching glutamatergic and inhibitory GABAergic connections (violet). **(D)** An “all” connection from cerebellum to PAG, reaching all PAG populations (light gray), PC, Purkinje cells; IO, Inferior olive; MCN, Medial cerebellar nuclei. **(E)** Schematic summary of models tested, source areas are connected to either glut (Model #1), glut+GABA (Model #2) or all other excitatory and inhibitory connections (Model #3 and 4) [same key color as panel **(A)**]. Model #5 represents the null hypothesis.

In this study, DCMs implemented for EEG/LFP data were adapted from the existing neuronal models (Kiebel et al., 2008; Moran R. et al., 2013) to reflect cerebellar and vlPAG neuronal circuitry. The implemented model (ERP neural mass model) reflects intrinsic excitatory and inhibitory projections between neuronal classes within one brain region. In the original neuronal assembly model this is represented by pyramidal cells which receive inhibitory and excitatory projections from local interneurons (both excitatory and inhibitory) which then project to other brain areas through extrinsic excitatory projections (see Figure 2 for the representation of the adapted models).

A DCM is defined by equations that summarize the synaptic dynamics of neuronal populations, by defining current and voltage changes. Each subpopulation of neurons is defined by a different set of dynamic equations (Eq. 1) that reflect the properties of that neuronal type defining the time-evolution of the neuronal post-synaptic potentials (*V*) as a convolution (⊗) of pre-synaptic firing rates (*S*) with a post-synaptic activation kernel *p(t)*.


(1) V=p⁢(t)⊗S

To simulate the dynamic membrane potentials resulting from the presence of ion-channels (Eq. 2), the convolution (*p*) of the average pre-synaptic firing input (*S*) on a group of synapses is obtained to transform the average density of pre-synaptic inputs (*S*) into average post-synaptic membrane potential (*V)* (David and Friston, 2003), where the convolution kernel is given by the following operator:


(2) $$p\,\left(t\right)=A\,\frac{H_{e/i}}{\tau_{e/i}}\,t\,e\,x\,p\,\left(\frac{-t}{\tau_{e/i}}\right)$$

*H*_*e/i*_ controls the maximum post-synaptic potential and τ_e/i_ the time constant of either excitatory (e) or inhibitory (i) receptors (time constants are for the main AMPA—8 ms and GABA_a_—16 ms receptor types for excitatory and inhibitory synapses, respectively) and account for receptor opening times. We denote connections as “*A*” parameters. If the synapse is not effective, the model should return a value of *A* close to zero. If the synapse is effective, then *A* should be non-zero.

Finally, to close the loop from input to output to input, the membrane potentials of each subpopulation (*V*) are then transformed into firing rate of this post-synaptic population (S, Eq. 3), and represents the input to other downstream subpopulations. *S* is represented as a sigmoid nonlinearity:


(3) $$S\,\left(V\right)=\frac{1}{1+\text{exp}\,\left(-r\,V\right)}-\frac{1}{2}$$

In this operator, *r* is a fixed parameter that controls the curvature (*r* = 0.56). *A priori* inter-regional conduction delays of 2 ms (delay within a region) and 16 ms (delays from vlPAG to MCN and MCN to vlPAG) complete the model specification. Parameters are optimized around a prior distribution with a Gaussian density—specified by a mean and variance (Table 1).

**TABLE 1:** Dynamic causal model (DCM) specification.

Specification (DCM.options)	Description	Parameter used
**Common parameters**		
Analysis	Data feature to be modeled	ERP
Model	Type of neural mass model	ERP
Spatial	Type of spatial (forward) model	LFP
Trials	Indices of trials (conditions)	1
Tdcm	Time window in ms (start, end)	(1, 200)
Onset	Stimulus onset in ms (component 1, component 2)	(10, 40)
Dur	Stimulus dispersion (standard deviation) in ms (component 1, component 2)	(8, 8)
τ	Receptor time constant (excitatory, inhibitory)	(8, 16)
**Distinct model parameters**		
“Glu”; “IO”	Extrinsic rates, region to region connectivity	1
“Glu-GABA”	Extrinsic rates, region to region connectivity	1/2
“All”	Extrinsic rates, region to region connectivity	1/8

List of the specifications and their description used in this study. Trial was set as 1 as each trial block was computed on a separate DCM. The time window (Tdcm) was set to include the time window of the main ERP component. The onset parameter estimates when the stimulus (in this case tone offset at time 0) might be activating the areas of interest, two values were selected to reflect the two deflections found in the ERPs. The stimulus dispersion provides an indication of the standard deviation of the estimated onset.

For further detail on the DCM development (see Jansen and Rit, 1995; David and Friston, 2003; David et al., 2005, 2006; Kiebel et al., 2007).

### DCM implementation and specification

Dynamic causal models are available in the “Statistical Parametric Mapping” (SPM)^1^ toolbox on MATLAB.

In this case the “spm_dcm_erp.m” function was used to model the ERPs. This function requires the user to define specific parameters that will provide information on the characteristic of the neural responses under investigation.

**FIGURE 3:**
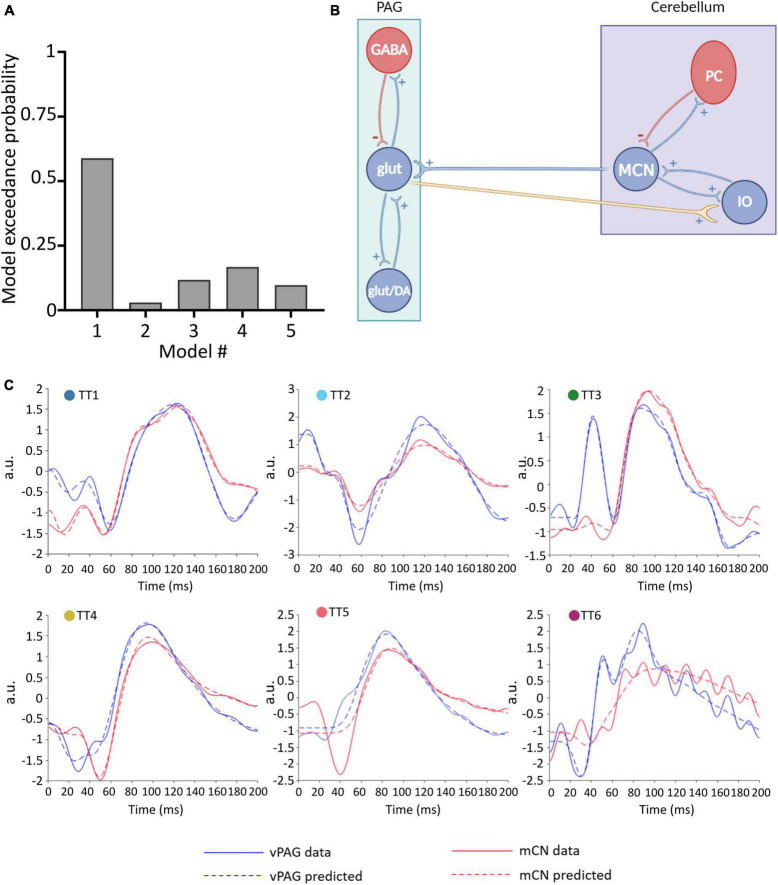
Winning neural model of MCN-vlPAG connectivity. **(A)** The model probability for each model tested, obtained from BMS. **(B)** Schematic representation of the winning model (model #1) showing excitatory extrinsic projections from the PAG to the cerebellum. **(C)** For each animal (TT1-6) the predicted ERP (dashed lines) using Model 1 is plotted in comparison with the real ERP data (filled lines) for the vlPAG (blue) and MCN (red).

The specifications used in this study are reported in Table 1, and were chosen as they provide the best fit of the data, i.e., the one that fitted most closely the model curve to the experimental ERP data (shown in Figure 3C).

The dataset consisted of five blocks from extinction (each consistent of an average of seven trials) that were computed separately. For each model, a matrix showing the changes in the strength of coupling between areas was defined with connections from cerebellum to vlPAG and from vlPAG to cerebellum both switched on. Finally, experimental inputs on connections were defined with all inputs were switched on, indicating that the stimulus (the offset of the tone) may influence both regions.

Connectivity models were built based on current literature on the cytoarchitecture of the cerebellum and vlPAG: (1) projections from the vlPAG to the cerebellum were tested as “IO” projections (glutamatergic), as based on previous studies (Koutsikou et al., 2014) it is likely that projections from the vlPAG to the MCN pass via the inferior olive (IO; Figure 2A) (2) projections from the cerebellum to the vlPAG included multiple options, as studies from our group and others (Frontera et al., 2020; Vaaga et al., 2020; Lawrenson et al., 2022) have shown that MCN glutamatergic projecting neurons synapse to different classes of neurons in the vlPAG such as excitatory glutamatergic or inhibitory GABAergic neurons as well as modulatory dopaminergic neurons. Therefore three separate models were included for MCN-PAG connectivity: a connection from cerebellum to PAG reaching only an excitatory glutamatergic population (Figure 2B), a connection from cerebellum to PAG reaching glutamatergic and inhibitory GABAergic connections (Figure 2C), an “all” connection from cerebellum to PAG, reaching all PAG populations (Figure 2D), DCM does not currently model dopaminergic neurons behavior, therefore these were not modeled directly, but are likely to be involved in the dynamics between these areas and will be considered in the discussion.

### Bayesian model selection

Bayesian model selection (BMS) is used to test the best model among a set of different hypotheses about how a specific dataset was generated (Stephan et al., 2009). The inversion of DCMs gives the posterior estimates of intrinsic (within brain area) and extrinsic (between brain areas) connectivity and the marginal likelihood (or evidence) for that model.

The evidence for each model is then compared to select the best model. In this study BMS was used to (i) select between two neurobiological models, one with self-recurrent connection that enhances inhibitory interneurons activity (with ii-to-ii) and one without this connection (without ii-to-ii) [see Moran R. et al. (2013) for detailed differences between these models] (as both produced a good fit of the data) and (ii) select between models of extrinsic connectivity between cerebellum-PAG (in this case a null model was also added, this is the simplest model, with two parameters set with a mean of zero and a prior variance of zero meaning that cannot be optimized during model inversion). In both cases, the model with highest evidence is then selected as the best model.

A BMS random-effect analysis (RFX) was used in this paper, to consider that the best model could differ between subjects, that have an unknown population distribution.

### Parametric empirical bayes

Parametric Empirical Bayes (PEB) is a hierarchical statistical model over parameters that allows one to test hypotheses on behavioral covariates across animals. Here we used the method to investigate the relationship between connection strengths and freezing, as an alternative to classical statistical methods, as it accounts for uncertainty in parameter estimates (variance) using the full parameter posterior, allowing to look at group effects and also between-subjects differences. Moreover, PEB was particularly appropriate for this data set as it is a more sensitive statistical method for small group size data with high variance. In addition, it allows to test for the absence of null hypothesis, therefore allowing to check for the absence of an effect.

Parametric Empirical Bayesian uses the DCMs per animal (*n* = 6) and session (*n* = 5) (total of 30 models in this case). The “between subject” design matrix contains information of the commonalities across subjects and the effects of interest (covariates). In this case, covariates of group mean, trial in extinction block and freezing were included. The data were mean-centered so that the first regressor represents group mean effective connectivity. In the within-subjects design matrix it was instead defined that the connectivity matrix can receive between-subject effects. In other words, indicating that the strength of the connections between cerebellum and PAG is the effect of interest.

The evidence of the estimated parameters is compared by selecting full and reduced models in which the estimated parameters are switched either on or off (i.e., the models that take into account all, some or none of the covariates, see Figure 5B). Finally, significant changes in connections (when they have a posterior probability of being greater than 0.95) are identified using Bayesian Model Reduction (BMR), a tool used to prune parameters that do not contribute to the model evidence. For a more detailed description of this methods see Zeidman et al. (2019).

### Statistical analysis

Graphical figures were plotted either in MATLAB or GraphPad Prism and statistical analysis was performed using either GraphPad Prism or R software. One-way ANOVA was used to compare changes in the peak amplitude of the ERPs across different blocks of extinction. A linear mixed-effects model with random intercept was used to investigate the relationship between the strength of the connection and freezing. Diagrammatic figures were made on Inkscape and BioRender.

## Results

Analysis of neural recordings in the MCN and vlPAG during an auditory fear conditioning task (Figure 1A) revealed that these two brain areas reliably generate ERPs (see Figure 1D for the group average waveforms) time locked to the offset of the conditioned tone. The characteristics of these ERPs, such as onset latency and changes in peak to peak amplitude during extinction were first reported in Lawrenson et al. (2022, see their Figure 3). The focus of the current paper is to use the same dataset and apply DCMs to determine the dynamics of connectivity between MCN and vlPAG and its relationship to freezing behavior.

A Bayesian model selection (BMS) was performed to verify which was the best neural mass model between the with ii-to-ii and the without ii-to-ii, that were both found to produce a good fit of the dataset. The winning model was without ii-to-ii which obtained a 0.93 exceedance probability, indicating that there is 93% confidence that this model is better suited to represent our data.

Two adapted models were then created using the “without ii-to-ii model” to represent cerebellar and PAG circuits, while maintaining the existing DCM structure (Figure 2, as described in Section “Materials and methods”). These models were used to identify the most likely extrinsic (between-region) connectivity model to represent MCN-vlPAG dynamics (see Section “Materials and methods” for rationale of these models). Five models were tested (Figure 2E); Four models were based on different representations of MCN-PAG connectivity while the last was a null hypothesis model with no extrinsic connection and therefore no input between the MCN and vlPAG (note that this is the simplest model).

To define the winning model, for each model (1–5), a parameter estimation was performed and the obtained DCMs were then placed in a BMS-RFX. Model 1 (Figure 3B) had the highest exceedance probability (0.59; Figure 3A), and therefore we selected this to continue the investigation of connectivity and behavior.

Model 1 includes bidirectional glutamatergic (i.e., excitatory only) connections (Figure 3B). In summary, the proposed circuitry between MCN and vlPAG assumes that: MCN has an excitatory projection to glutamatergic vlPAG neurons (and/or dopaminergic neurons, but this is not possible to test with the current models), while vlPAG projection neurons send excitatory input to the cerebellar nuclei neurons via the inferior olive (IO). The model provides a good fit for the majority of individual responses, predicting ERPs in both the MCN and vlPAG (Figure 3C). In one case (TT5) the MCN response was modeled less well, suggesting that in this specific case a different neuronal architecture may have been involved. Indeed, in this animal, histological reconstruction of the recording electrode tip position revealed a more dorsal position in the cerebellum compared to the other cases (see Figure 1B).

Reassuringly, the model was able to distinguish between 50 Hz electrical noise and biological response, for example in TT6 (Figure 3C) the predicted response was smoothed to follow the ERP waveform and not the noise related signal. This provides us with confidence that the model is indeed fitting the biological response.

**FIGURE 4:**
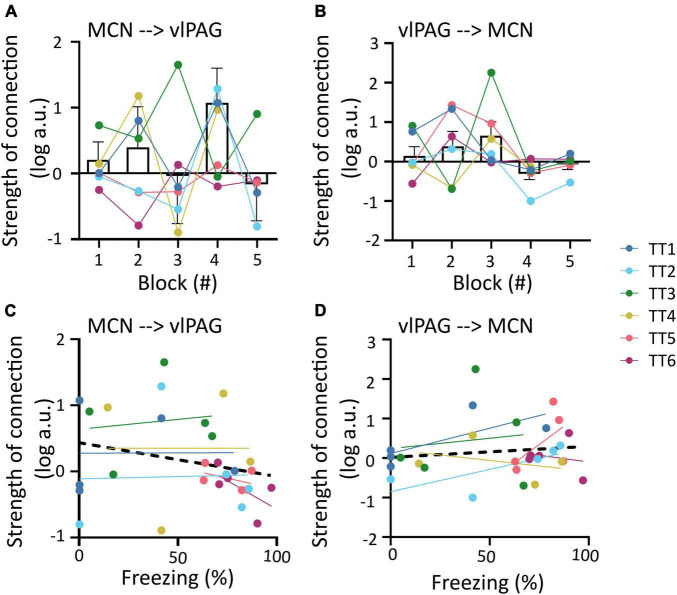
Changes in MCN-vlPAG coupling strength throughout extinction. **(A)** The strength of MCN-vlPAG connectivity during each trial block (1–5). Bar graph shows mean ± SEM, individual data points are shown in different colors for each animal. **(B)** Same as A but for vlPAG-MCN connectivity. **(C)** Correlation between strength of connection and freezing percentage for the MCN-vlPAG pathway, data points shown in different color for each animal, black line shows overall linear regression of data. **(D)** Same as C but for PAG-MCN pathway. No statistical difference was found in any of the comparisons shown in this figure.

The outputs from the DCMs of model 1 were then used to investigate changes in the strength of coupling between trial blocks for each direction of the connection (MCN to vlPAG versus vlPAG to MCN). For the MCN to vlPAG projection, an initial increase in strength of the connections in blocks 1, 2 was observed followed by a decrease in most animals and a subsequent increase at block 4 (by which most animals have extinguished fear), in block 5 there was again a reduction in strength of the connection. However, no significant statistical difference was found between the trial blocks [Figure 4A; *F*(1.393,6.619) = 0.8398, *p* = 0.432, mixed-effects analysis]. The strength of the vlPAG to MCN coupling, was more linear, showing a gradual increase of strength of connection. This was observed from trials 1–3, with a sudden decrease in trial 4 and 5, but there was no significant difference [Figure 4B; *F*(1.690,10.14) = 1.919, *p* = 0.198, mixed-effects analysis].

**FIGURE 5:**
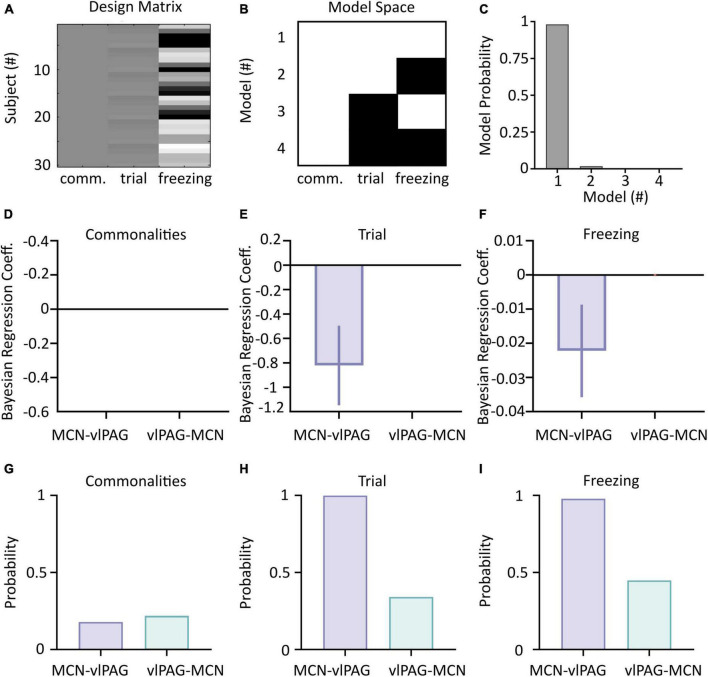
Parametric Empirical Bayesian (PEB) analysis of cerebellar-vlPAG connectivity during extinction. **(A)** The design matrix used to compute PEB. Each line is a different combination of animal/trial; column 1 represents the commonalities (group mean), column 2 represents the trials and column 3 represents the corresponding freezing levels. **(B)** Connectivity matrix indicating the models used in the BMS. A white square indicates that the parameter was included (on), while black square indicate that the parameter was excluded (off). **(C)** Model probability for each of the models shown in panel **(B)** shows that both trial and freezing effects (Second level Model 1) were present in changes in connectivity. **(D–F)** Bayesian Regression Coefficient of commonalities, trial and freezing on CB-PAG and PAG-CB connections; values of 0 indicate no statistical influence of the parameters on the changes in connectivity. **(G–I)** Posterior probabilities for CB-PAG and PAG-CB connections for commonalities, trial and freezing effect.

In addition we tested if the strength of the coupling between these regions correlated to freezing behavior. In both cases there was no significant correlation between the two variables (MCN-vlPAG; Figure 4C; *R*^2^ = 0.061, *p* = 0.194; vlPAG-MCN; Figure 4D; *R*^2^ = 0.016, *p* = 0.511; Pearson’s correlation).

### PEB analysis

To address the variance (noise of the data) amongst the subjects, another statistical method was used to assess dependencies between covariates: PEB, that allows for more precise estimates compared to classical methods.

Parametric Empirical Bayesian uses DCM output modeled as commonalities between subjects and takes into account trial number and freezing levels as covariates (Figure 5A). A comparison of reduced and full models (see Section “Materials and methods” and Figure 5B) is then used to identify which model best represents the data (Figures 5A–C).

Application of PEB to the dataset revealed that the full model (that accounts for the influence of both trial number and freezing levels on the neural data) was the best performing (98% model probability, Figure 5C), demonstrating that both trials and freezing parameters are needed to explain the group effects.

The winning full model was used to determine the influence of each covariate (trial number and freezing) on the two neural pathways (i.e., MCN- vlPAG versus vlPAG to MCN). While neither direction better accounted for the commonalities (strength of connections, Figures 5D, G), the MCN-vlPAG projection had higher probability of explaining changes due to trial number and freezing (Figures 5H, I). Examination of the effect size showed that the MCN-vlPAG pathway had a negative effect of trial and freezing, i.e., higher connectivity strength of MCN-vlPAG would lead to reduced freezing behavior (Figures 5E, F), reproducing the trend seen with the correlation analysis (Figure 4C).

These results are in agreement with our hypothesis that offset signals are a correlate of predictive processes and are modulated by the MCN-vlPAG pathway during extinction of conditioned freezing.

## Discussion

While it is now widely accepted that the cerebellum contributes to survival circuits, and is involved in the acquisition, consolidation and extinction of fear processes, the underlying neural activity and mechanisms that encodes these adaptive changes is still unclear. Here we propose that the cerebellum supports extinction to reduce the conditioned fear memory by increasing its connectivity with the vlPAG, time locked to the offset of a conditioned tone; where a prior US (footshock) has been paired with a predictive CS (auditory cue).

We show that DCMs can be reliably used to study the connectivity of rodent ERP signals from subcortical structures to investigate the relationship between changes in coupling and behavioral outputs. In particular, we have shown that MCN-vlPAG pathway connectivity changes over the time course of extinction, reflected by changes in freezing behavior. Lower freezing levels are paralleled by increases in coupling between these two regions, suggesting that the communication between the cerebellum and vlPAG might be important in the extinction of conditioned freezing. The role of the PAG has been extensively studied in defensive states and many neural signatures encoding fear related processes have been identified. Our latest study (Lawrenson et al., 2022) and other groups have identified neural responses at tone onset and related such activity to multiple aspects of fear processing, such as maintenance of an aversive memory (Watson et al., 2016), prediction error (Johansen et al., 2010; Ozawa et al., 2017) and threat probability (Wright and McDannald, 2019). By comparison, responses to a conditioned tone offset within the vlPAG and MCN, are much less well described, although such responses have been observed in other brain regions (Quirk et al., 1997; Kim et al., 2015; Liu et al., 2019). As such, the functional and behavioral role of such a response is yet to be determined.

Several studies (Koutsikou et al., 2014; Cacciola et al., 2019; Frontera et al., 2020; Vaaga et al., 2020; Lawrenson et al., 2022) have investigated the interactions between the cerebellum and the PAG (a key area in the regulation of defensive behaviors), and have shown that pathways connecting these two areas participate in the expression of fear behaviors, during both acquisition and extinction of aversive stimuli. None of these studies have found evidence that modulation of the MCN-vlPAG pathway changes general motor coordination, suggesting that changes in freezing behavior are related to defensive state. Together with behavioral changes during early extinction (recall of the fear memory), we previously found that the vlPAG offset responses were affected by cerebellar manipulation (Lawrenson et al., 2022). To further investigate if MCN-vlPAG connectivity reduces conditioned fear during extinction, we applied DCM to the ERPs at tone offset recorded from the cerebellum and PAG.

This allowed us to infer that the strength of the coupling between the cerebellum and vlPAG is inversely related to freezing behavior. We have reported results acquired both with classical statistics and PEB, although classical statistics did not show a significant effect, the PEB regression coefficient was high. We believe that PEB is the most suited statistical approach for this specific data set. In fact, PEB allows to identify differences with small group size data that has high variance (noisy estimates); it allows us also to test for the absence of the hypothesis, therefore enabling to check for the absence of an effect together with the presence of it. The results reported with PEB are consistent with behavioral findings from our group (Lawrenson et al., 2022), where an inhibition of the pathway resulted in lower rates of extinction (higher freezing levels for a prolonged time). Our results are also consistent with Frontera et al. (2020) who provided evidence in mice that chemogenetic inhibition of the pathway during extinction increases freezing levels, while optogenetic activation decreases them. The model here presented provides for a causal analysis of information flow using a plausible biophysical model, that cannot be assumed by correlating units with behavior. In associatively conditioned fear learning, prediction error signals play an important role in the acquisition and extinction of fear memories. During the initial stages of unreinforced extinction training the conditioned tone predicts an aversive shock when none occurs, resulting in a negative prediction error. Offset responses are present during these early extinction trials. Repeated omission of the aversive shock results in extinction learning in which the tone no longer predicts the occurrence of the aversive shock (instead predicting its absence). Offset responses decrease in amplitude during this extinction learning process. Therefore, tone offset responses could be representative of predictive signals that support conditioned behavior and their absence may contribute to fear extinction processes. Although the current study has allowed us to explore the dynamics between MCN and vlPAG by relying on neurobiologically motivated models, there are some limitations on the use of DCM in this context. First, Vaaga et al. (2020) has shown that although the MCN targets glutamatergic and GABAergic neurons, it also targets a high percentage of dopaminergic neurons in the vlPAG. Studies *in vitro* demonstrated that activation of the MCN projection onto dopaminergic neurons favors inhibition of glutamatergic neurons that are known to directly regulate freezing behavior (Vaaga et al., 2020), leading them to the conclusion that suppression of cerebellar activity may facilitate freezing which is consistent with our hypothesis. This suggests that the mechanisms modeled here with glutamatergic and GABAergic neurons might also require dopaminergic activity. In support of the hypothesis that offset responses might encode predictive signals, dopaminergic neurons were previously shown by Groessl et al. (2018) to encode a positive prediction error in the vlPAG (although in this case the response was likely to be related to both shock and offset response). The influence of dopaminergic activity might still be implicitly captured in these models, but is not accounted for directly as neuromodulators such as dopamine operate at different (slower) time scales that cannot be captured in the DCM biophysical models. Nonetheless the overall synaptic gain that is represented in the current model can still represent the modulation described by Vaaga et al. (2020) and that regulates the final freezing behavior.

Another important consideration is that the neuronal models used did not reflect the detailed structure of the brain regions under study. For example, the current cerebellar model lacks an excitatory climbing fiber projection from the inferior olive to Purkinje cells. Nonetheless, the models fitted the dataset very well, presumably because they were able to reflect the overall balance of intrinsic excitatory and inhibitory connections on the output neuronal populations.

Despite these potential limitations, the present study presents a first interpretation of the neural mechanisms that underpin cerebellar-PAG pathway modulation of fear behaviors, using ERPs and DCM. Offset responses likely represent an important neural feature in fear circuits and could be a key to understanding how fear extinction is regulated.

## Data availability statement

The data analyzed in this study is subject to the following licenses/restrictions: The dataset is not currently publicly available as still under analysis by the owner. Requests to access these datasets should be directed to EP, elena.paci@bristol.ac.uk and CL, pycll@bristol.ac.uk.

## Ethics statement

The animal study was reviewed and approved by the University of Bristol Animal Welfare and Ethical Review Body.

## Author contributions

EP, RM, CL, RA, and BL contributed to the conception and design of the study. CL collected the dataset. EP and RM performed the analysis. EP wrote the first draft of the manuscript. All authors contributed to manuscript editing and revision, read, and approved the submitted version.

## Footnotes

1
https://www.fil.ion.ucl.ac.uk/spm/
